# Laparoscopic versus Open Total Gastrectomy for Gastric Cancer: An Updated Meta-Analysis

**DOI:** 10.1371/journal.pone.0088753

**Published:** 2014-02-18

**Authors:** Weizhi Wang, Xiaoyu Zhang, Chen Shen, Xiaofei Zhi, Baolin Wang, Zekuan Xu

**Affiliations:** 1 Department of General Surgery, The First Affiliated Hospital of Nanjing Medical University, Nanjing, China; 2 Department of General Surgery, The Second Affiliated Hospital of Nanjing Medical University, Nanjing, China; University Hospital Heidelberg, Germany

## Abstract

**Objective:**

To expand the current knowledge on the feasibility and safety of laparoscopic total gastrectomy (LTG) for gastric cancer in comparison with open total gastrectomy (OTG).

**Background:**

Additional studies comparing laparoscopic versus open total gastric resection have been published, and it is necessary to update the meta-analysis of this subject.

**Methods:**

Original articles compared LTG and OTG for gastric cancer, which published in English from January 1990 to July 2013 were searched in PubMed, Embase, and Web of Knowledge by two reviewers independently. Operative time, blood loss, harvested lymph nodes, proximal resection margin, analgesic medication, first flatus day, first oral intake, postoperative hospital stay, postoperative complications, hospital mortality, 5-year overall survival (OS) and disease-free survival (DFS) were compared using STATA version 10.1.

**Results:**

17 studies were selected in this analysis, which included a total of 2313 patients (955 in LTG and 1358 in OTG). LTG showed longer operative time, less blood loss, fewer analgesic uses, earlier passage of flatus, quicker resumption of oral intake, earlier hospital discharge, and reduced postoperative morbidity. The number of harvested lymph nodes, proximal resection margin, hospital mortality, 5-year OS and DFS were similar.

**Conclusion:**

LTG had the benefits of less blood loss, less postoperative pain, quicker bowel function recovery, shorter hospital stay and lower postoperative morbidity, at the price of longer operative time. There were no statistical differences in lymph node dissection, resection margin, hospital mortality, and long-term outcomes, which indicated the similar oncological safety with OTG. A positive trend was indicated towards LTG. So LTG can be performed as an alternative to OTG by the experienced surgeons in high-volume centers. Whereas, due to the relative small sample size of long-term outcomes and lack of randomized control trials, more studies are required.

## Introduction

Since the first laparoscopic gastrectomy for gastric caner was performed by Japanese surgeons in 1991 [1], laparoscopic distal gastrectomy (LDG) for early gastric cancer has gained widely acceptance for its minimal invasion compared with open distal gastrectomy (ODG). Many studies have demonstrated the benefits of LDG over open surgery, such as less blood loss, shorter hospital stay, accelerated recovery, extended lymphadenectomy and reduced postoperative complications [2]–[6]. Moreover, the indications for LDG even extended from early gastric cancer to advanced gastric cancer [7]–[10]. However, limited surgeons chose laparoscopic total gastrectomy (LTG) instead of open total gastrectomy (OTG) for proximal or middle-third gastric cancer due to the technical difficulties in sufficient lymph node dissection, vascular procedures along the greater curvature of the proximal stomach and the performance of esophagojejunostomy. With the development of the laparoscopic instruments and the increasing experiences in complex gastric procedures, the use of LTG is increasing annually. Several studies have reported the use of LTG as the treatment of gastric cancer and indicate its potential superiority [11], [12]. Recently, a meta-analysis published by Haverkamp *et al.* also demonstrated the better short-term outcomes of LTG compared with OTG [13]. Nevertheless, only eight studies were involved in this meta-analysis and most of them focused on the early gastric cancer with a small sample size, lacking long-term outcomes. Thus, the feasibility and safety of LTG are still needed further validation. Because seven additional studies which contain more cases of advanced gastric cancer and more survival data, have now been published, we thought to perform an updated meta-analysis to broaden the current knowledge on the feasibility and safety of LTG for gastric cancer.

## Materials and Methods

### Literature search

Literatures that published in English from January 1990 to July 2013 were searched in the following database: PubMed, Embase, and Web of Knowledge. The keywords “laparoscopic”, “total gastrectomy”, “gastric cancer”, “randomized controlled trial”, “prospective study”, and “comparative study” were used. Then, all titles, abstracts, or related citations were scanned and reviewed.

### Inclusion and exclusion criteria

Inclusion criteria were described as follow: (1) studies that compared LTG with OTG for gastric cancer; (2) LTG that was performed with either laparoscopy-assisted or total laparoscopic approach; (3) any type of comparative study; (4) studies with any size.

Exclusion criteria were used as follows: (1) studies including other types of gastric resection, unless the data were presented separately; (2) studies in which <3 interested indexes were reported, or the indexes were difficult to calculate from the results; (3) overlapping data.

### Quality assessment of the studies

Newcastle–Ottawa Quality Assessment Scale for cohort studies (NOS) (Table 1) [14], which is recommended in the Cochrane Handbook version 5.1.0, was used to evaluate the quality of the nonrandomized studies by two independent reviewers. Eight elements in this scale are used to assess patient population and selection, study comparability, follow-up, and outcome of interest. High-quality elements are awarded by adding a star, and then the stars are added up to compare the study quality. Each study was graded as either low quality (0–5) or high quality (6–9). The results were presented in Table 2, and the low-quality studies were excluded. Any discrepancies were resolved by a consensus reviewer.

**Table 1:** Newcastle–Ottawa quality assessment scale*.

**Selection**
(1) Representativeness of the exposed cohort
(a) Truly representative of the average ‘GC patient’ in the community (1 star)
(b) Somewhat representative of the average ‘GC patient’ in the community (1 star)
(c) Selected group of users (e.g. nurses, volunteers)
(d) No description of the derivation of the cohort
(2) Selection of the non-exposed cohort
(a) Drawn from the same community as the exposed cohort (1 star)
(b) Drawn from a different source
(c) No description of the derivation of the non-exposed cohort
(3) Ascertainment of exposure
(a) Secure record (e.g. surgical records) (1 star)
(b) Structured interview (1 star)
(c) Written self-report
(d) No description
(4) Demonstration that outcome of interest was not present at start of study
(a) Yes (1 star)o
(b) No
**Comparability**
(1) Comparability of cohorts on the basis of the design or analysis
(a) Study controls for ‘ age, sex, BMI’ (1 star)
(b) Study controls for any additional factor (1 star) (ASA, tumor size, stage etc.)
**Outcome**
(1) Assessment of outcome
(a) Independent blind assessment (1 star)
(b) Record linkage (1 star)
(c) Self-report
(d) No description
(2) Was follow-up long enough for outcomes to occur?
(a) Yes (‘2 years’) (1 star)
(b) No
(3) Adequacy of follow-up of cohorts
(a) Complete follow-up – all subjects accounted for (1 star)
(b) Subjects lost to follow-up unlikely to introduce bias – small number lost ‘5%’ or description provided of those lost (1 star)
(c) Follow-up rate ‘>95%’ and no description of those lost
(d) No statement

^*^A study can be awarded a maximum of one star for each numbered item within the Selection and Outcome categories. A maximum of two stars can be given for Comparability. Underlined and quoted phrases are provided in the scale to allow for adjustment to particular studies. Italicized phrases indicate our interpretation of the question relevant to this study.

GC, gastric cancer; ASA, American Society of Anesthesiology classification; BMI, body mass index.

**Table 2:** Assessment of Quality of Studies.

	selection				comparability	outcome			
References	1	2	3	4	5	6	7	8	score
Dulucq et al [22]		*	*	*	*	*	*	*	7
Usui et al [23]	*	*	*	*	**	*			7
Mochiki et al [24]	*	*	*	*		*	*	*	7
Kim et al [25]	*	*	*	*	*	*			6
Topal et al [26]	*	*	*	*	**	*			7
Kawamura et al [27]	*	*	*	*	**				6
Sakuramoto et al [28]	*	*	*	*	*	*	*	*	8
Du et al [29]	*	*	*	*	**	*	*	*	9
Kim et al [30]	*	*	*	*	**	*			7
Eom et al [31]	*	*	*	*	*	*	*	*	8
Amanda K. et al [31]	*	*	*	*		*			5
Kunisaki et al [33]	*	*	*	*	**	*	*		7
Siani et al [34]	*	*	*	*	*		*		6
Jeong et al [35]	*	*	*	*	**	*			7
Guan et al [36]	*	*	*	*	*	*			6
Lee et al [37]	*	*	*	*	**	*	*	*	9
Kim et al [38]	*	*	*	*	**	*			7
Bo et al [39]	*	*	*	*	**	*	*	*	9

### Methods of review

The data was extracted and critically appraised independently by two authors. We extracted operative time, blood loss, number of harvested lymph nodes, and proximal resection margin to assess the effectiveness of the procedures. The analgesic medication, first flatus day, first oral intake and hospital stay were used to compare the postoperative recovery of the procedures. The postoperative complications including wound infection, anastomotic leakage, anastomotic stenosis, postoperative ileus, pneumonia, pancreatitis, intra-abdominal abscess and adhesive bowel obstruction were compared. The hospital mortality, 5-year overall survival (OS) and disease free survival (DFS) were used to estimate the postoperative safety of LTG.

### Statistical analysis

We used weighted mean differences (WMD) with 95% confidence intervals (CI) to analyze continuous variables presented in the same scale (i.e., operative time, blood loss, postoperative hospital stay). When a study reported a range instead of standard deviation (SD), a quarter of the range amplitude was equivalent to the estimated SD [15]. Dichotomous data was calculated by relative risks (RR). OS and DFS were evaluated by pooled hazard ratios (HR) and their 95% confidence intervals (CI). HRs and their 95% CIs for OS and DFS were obtained from the original study. If the study did not directly report the HR and 95% CI, we used the published methods to calculate them [16], [17]. Plot Digitizer version 2.6.3 was used to read Kaplan-Meier curves of the included studies (free software downloaded from http://plot-digitizer.softpedia.com/). HR calculation spreadsheet was used to calculate the HR and 95% CI (freely downloaded from http://www.trialsjournal.com/content/supplementary/1745-6215-8-16-s1.xls). Random effects models were used owing to the high heterogeneity of the studies, otherwise, fixed-effects models were used [18], [19]. We used the Cochran's *Q* – test to assess heterogeneity, and *P*<0.1 was considered significant. Galbraith plot was used to identify the major contributors to heterogeneity [20]. And in order to explore the heterogeneity among the outcome variables better, we examined study quality (<8 and ≥8), year of publication (before and after 2012), country of patients (eastern and western), sample size (<50 and ≥50), stage of gastric cancer (early, advanced and both) and lymph nodes dissection (D1, D2 and both) in meta-regression model. Subgroup analyses according to the meta-regression results and study characteristics were performed to explore the possible explanations of the heterogeneity and to assess the potential effect of these factors on outcomes. Funnel plots and Egger's linear regression test were used to assess the publication bias [21]. All statistical calculations were completed by using STATA (version 10.1, StataCorp LP, College Station, TX). A two-trailed value of *P*<0.05 was considered significant.

## Results

### Search results

**Figure 1:**
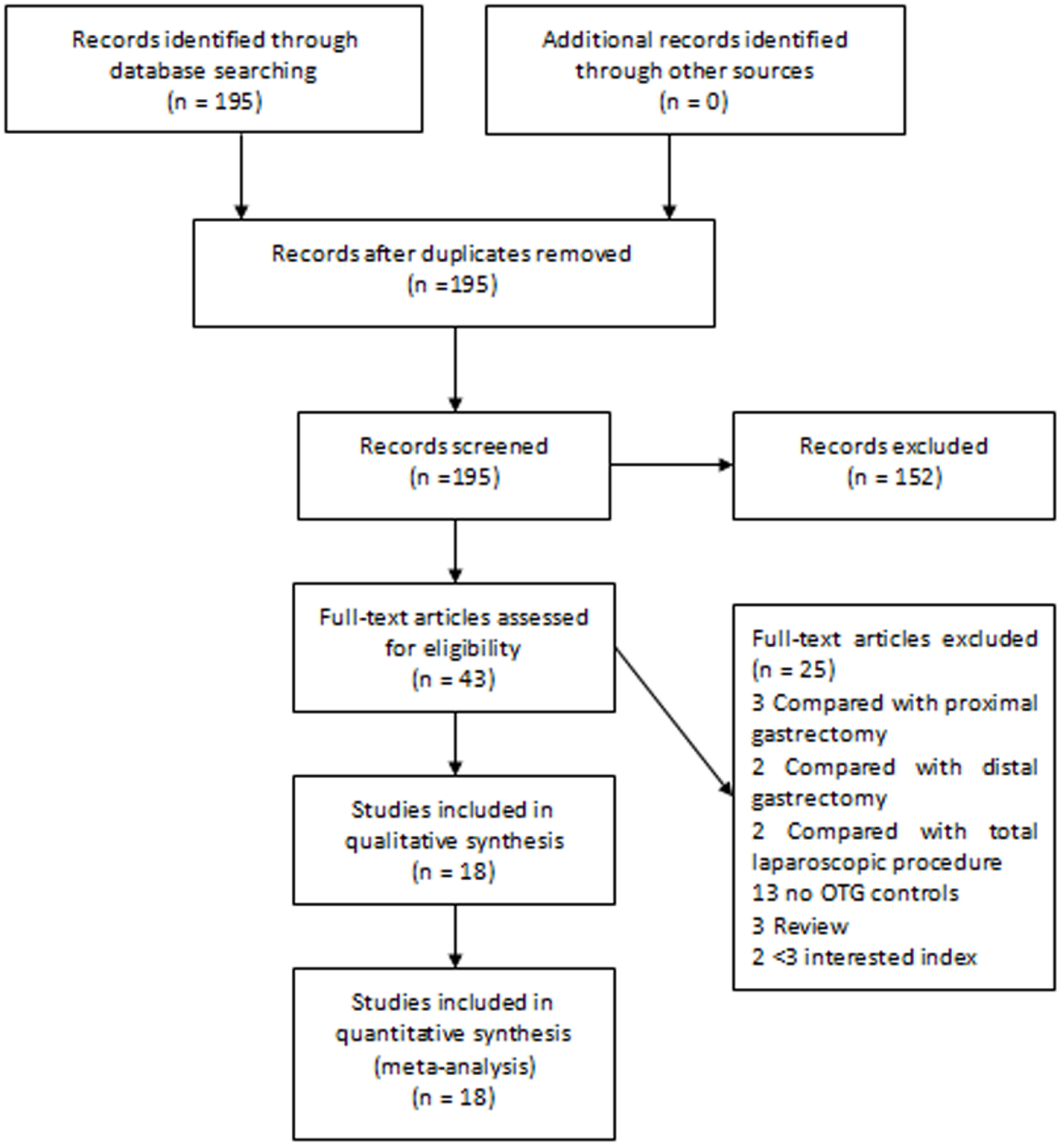
Articles identified with criteria for inclusion and exclusion.

The electronic search strategy identified 195 articles that mentioned laparoscopic gastrectomy and open gastrectomy for gastric cancer. After screening the titles, abstracts, full texts, or a combination of these, we selected articles based on the inclusion and exclusion criteria (Fig 1). Finally, 18 articles that compared LTG with OTG were included [22]–[39]. Then the NOS was used to assess the quality of each study and one study was excluded for its low scores [32]. Finally, 17 studies were selected for this analysis (Table 3). In Haverkamp *et al.*'s study [13], two articles were regarded as contributor to high risk of bias [23], [33] and were excluded at last. But in our assessment, these two studies were graded as high quality and were included in our meta-analysis.

**Table 3:** Details of the articles included in the meta-analysis.

				Sample size		
References	Year	Country	Journal	LTG	OTG	Type of the study
Dulucq et al [22]	2005	France	Surg Endosc	8	11	Prospective Cohort study
Usui et al [23]	2005	Japan	SURG LAPARO ENDO PER	20	19	Cohort study
Mochiki et al [24]	2008	Japan	Surg Endosc	20	18	Cohort study
Kim et al [25]	2008	South Korea	J LAPAROENDOSC ADV S	27	33	Prospective Cohort study
Topal et al [26]	2008	Belgium	Surg Endosc	38	22	Prospective Cohort study
Kawamura et al [27]	2009	Japan	World J Surg	46	35	Cohort study
Sakuramoto et al [28]	2009	Japan	Surg Endosc	30	44	Cohort study
Du et al [29]	2010	China	Hepato-Gastroenterology	82	94	Retrospective Cohort study
Kim et al [30]	2011	South Korea	J Korean Surg Soc	63	127	Retrospective Cohort study
Eom et al [31]	2012	South Korea	Surg Endosc	100	348	Case-control Cohort study
Kunisaki et al [33]	2012	Japan	Surg Endosc	27	30	Prospective Cohort study
Siani et al [34]	2012	Italy	MINERVA CHIR	25	25	Retrospective Cohort study
Jeong et al [35]	2012	South Korea	J Am Coll Surg	122	122	Prospective Cohort study
Guan et al [36]	2012	China	Surg Endosc	41	56	Case-control Cohort study
Lee et al [37]	2013	South Korea	Surg Endosc	50	50	Prospective Cohort study
Kim et al [38]	2013	South Korea	J LAPAROENDOSC ADV S	139	207	Prospective Cohort study
Bo et al [39]	2013	China	J Gastrointest Surg	117	117	Case-control Cohort study

LTG, laparoscopic total gastrectomy; OTG, open total gastrectomy.

### Characteristics of the studies

All the articles were published between 2005 and 2013. A total of 2313 patients were involved in the meta-analysis, which contained 955 people undergoing LTG and 1358 people receiving OTG. Fourteen studies were published by Asian investigators, and only three were reported by the western scholars. This result can be explained by the high incidence of gastric cancer in eastern countries. And the conclusions might bias to the Asians. As shown in Table 4, five articles reported the treatment for patients with early gastric cancer (EGC) [23], [24], [27], [30], [31], and three studies focused on the patients with advanced gastric cancer (AGC) [29], [38], [39]. The rest of the nine articles described both populations [22], [25], [26], [28], [33]–[37]. The mean age of the studies ranged from 50 to 75 years, and distributed similarly in the two procedures. The frequency of the gender was also found distributing equally in the two groups. A similar mean BMI between 22 and 24 kg/m^2^ was observed in most studies expect one [31], however, when we pooled the data together, the patients in LTG group was found having a lower mean BMI (WMD, −0.32; 95% CI, −0.62, −0,01; *P* = 0.041). We also compared the tumor size. Apart from two studies that reported the statistical difference [24], [28], the rest six showed no significant difference [22], [26], [29]–[31], [38]. What's more, all studies described the similar distribution of pTNM stages and ASA scores except two [28], [31].

**Table 4:** Characteristics of the articles included in the meta-analysis.

References	Approach	Age (years)	Male(No.)	BMI (kg/m^2^)	Tumor diameters (cm)	Lymph nodes dissection	Population	ASA (No.)			Stage (No.)			
								1	2	3	I	II	III	IV
Dulucq et al [22]	LTG	75±8	3		5.5±2	D1	EGC+AGC							
	OTG	67±14	5		6.1±0.4									
Usui et al [23]	LTG	66.0±10.4	13	21.3±3.1		D1	EGC							
	OTG	66.2±10.2	14	22.1±2.4										
Mochiki et al [24]	LTG	66±2.4	16		3.6±0.5	D1	EGC							
	OTG	63±2.2	16		5.7±0.8									
Kim et al [25]	LTG	57.3±14.2	16	22.6±3.1		D1, D2	EGC+AGC							
	OTG	61.6±9.2	23	22.4±2.1										
Topal et al [26]	LTG	68.0±12	23	24.0±3	4.7±4.3	D2	EGC+AGC				17	7	10	4
	OTG	69.0±12	17	24.0±3	3.0±4.3						7	7	6	2
Kawamura et al [27]	LTG	64.0±10.4	36	22.8±3.0		D2	EGC	15	27	4				
	OTG	65.2±10.7	25	22.9±2.4				14	15	6				
Sakuramoto et al [28]	LTG	63.7±9.2	12	21.9±2.7	4.0±2.9	D1, D2	EGC+AGC	9	20	1	25	2	3	0
	OTG	67.2±9.9	10	22.5±3.6	6.1±3.7			8	28	8	15	17	12	0
Du et al [29]	LTG	60.4±18.5	54	22.3±2.6	5.4±1.4	D2	AGC				3	36	42	0
	OTG	57.8±17.2	61	22.5±2.4	5.9±1.9						6	31	57	0
Kim et al [30]	LTG	55.9±12.2	43	22.7±2.5	3.8±2.1	D2	EGC	45	15	3				
	OTG	57.3±11.1	81	23.0±2.9	3.9±2.7			86	39	2				
Eom et al [31]	LTG	54.9±13.5	57	22.7±2.8	4.3±2.9	D2	EGC				100	0	0	0
	OTG	58.7±11.5	254	23.8±2.9	4.4±3.0						348	0	0	0
Kunisaki et al [33]	LTG	67.4±11.0	21	23.5±2.5		D1, D2	EGC+AGC	11	14	2				
	OTG	67.1±6.6	20	24.3±4.3				9	16	5				
Siani et al [34]	LTG	65±8.5	15			D1, D2	EGC+AGC				6	5	14	0
	OTG	66±7.8	18								4	5	16	0
Jeong et al [35]	LTG	63.2±11.2	89	23.1±3.4		D1, D2	EGC+AGC	33	80	9	105	13	4	0
	OTG	62.6±11.7	93	23.5±3.2				43	67	12	99	16	7	0
Guan et al [36]	LTG	60.7±9.1	33			D2	EGC+AGC				18	20	3	0
	OTG	57.8±9.9	40								25	25	6	0
Lee et al [37]	LTG	50.6±22.1	32	23.2±3.7		D2	EGC+AGC	85	46	8	24	13	9	4
	OTG	51.0±22.6	32	23.0±3.4				137	52	18	24	13	9	4
Kim et al [38]	LTG	58.0±13.5	86	23.6±4.7	3.2±3.7	D2	AGC				0	0	139	0
	OTG	56.0±13.3	134	24.1±4.6	4.0±5.4						0	0	207	0
Bo et al [39]	LTG	54.5±10.6	82	21.1±3.0		D2	AGC				6	40	52	19
	OTG	52.6±13.6	80	21.7±3.8							4	38	55	20

LTG, laparoscopic total gastrectomy; OTG, open total gastrectomy; EGC, early gastic cancer; AGC, advanced gastric cancer; BMI, body mass index; ASA, American Society of Anesthesiology classification.

D1 lymph node dissection of total gastrectomy, which requires the retrieval of lymph nodes along the left gastric artery and the common hepatic artery, around the celiac artery, was performed in three articles [22]–[24]. Modified D2 lymph node dissection of total gastrectomy (without pancreatectomy and splenectomy), which refers to the removal of additional lymph nodes around the splenic artery and hilus of the spleen, and those located in the hepatoduodenal ligament, was achieved in nine articles [26], [27], [29]–[31], [36]–[39]. In the rest five studies, both D1 and D2 dissection were used [25], [28], [33]–[35]. All the studies reported the performances of Roux-en-Y reconstruction and esophagojejunal anastomosis.

### Operative findings

**Figure 2:**
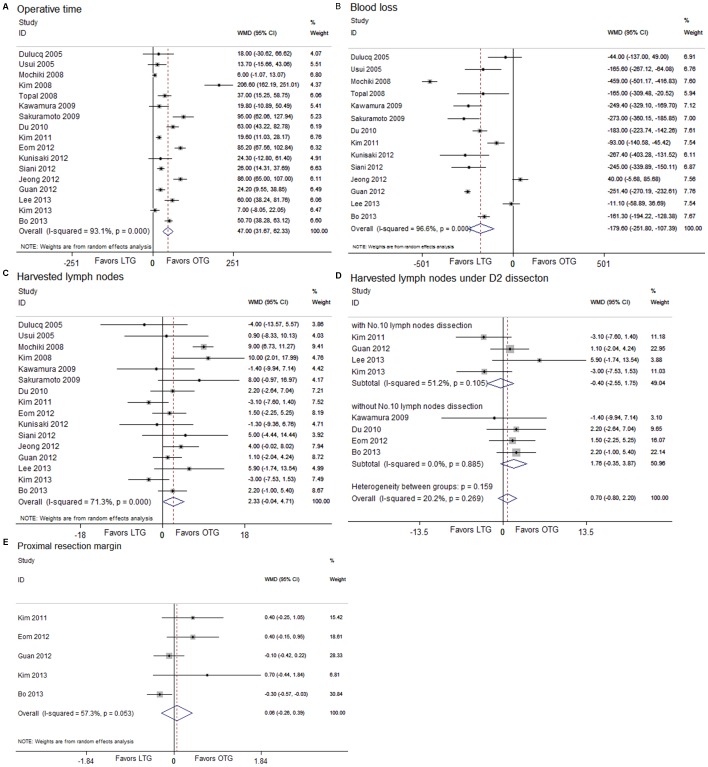
Analysis comparing (A) operative time, (B) blood loss, (C) harvested lymph nodes, (D) harvested lymph nodes under D2 dissection, and (E) proximal resection margin.

There was a longer duration of operative time in the LTG group than that in the OTG group (WMD, 47.00; 95% CI, 31.67, 62.33; *P*<0.001) (Fig 2A). However, significant heterogeneity (I^2^ = 93.1%, *P*<0.001) and the publication bias (t = 2.93; *P* = 0.010) were observed. Therefore, we used the Galbraith plot to find the source of heterogeneity [24], [25], [28], [29], [31], [35], [37]–[39], and excluded them all. Then, we meta-analyzed this subject again and still found the same outcome (WMD, 22.86; 95% CI, 17.18, 28.53; *P*<0.001) with low heterogeneity (I^2^<0.1%, *P* = 0.890) and no publication bias (t = 0.31; *P* = 0.764). Blood loss during the operation was decreased under the laparoscopic procedure (WMD, −179.60; 95% CI, −251.80, −107.89; *P*<0.001) (Fig 2B). No statistical difference was found between the two groups in the number of harvested lymph nodes (WMD, 2.33; 95% CI, −0.04, 4.71; *P* = 0.054) (Fig 2C). We also analyzed the retrieval of lymph nodes under the modified D2 lymphadenectomy between LTG and OTG, and no difference was discovered (WMD, 0.70; 95% CI, −0.80, 2.20; *P* = 0.361) (Fig 2D). Moreover, the effect of No. 10 lymph nodes dissection under D2 lymphadenectomy on the number of harvested lymph nodes was assessed, and no significant difference between the two groups was found (with No. 10 lymph nodes dissection: WMD, −0.40; 95% CI, −2.55, 1.75; *P* = 0.715; without No. 10 lymph nodes dissection: WMD, 1.76; 95% CI, −0.35, 3.87; *P* = 0.102). The length of the proximal resection margin was similar for either group (WMD, 0.06; 95% CI, −0.26, 0.39; *P* = 0.706) (Fig 2E).

### Postoperative clinical course

**Figure 3:**
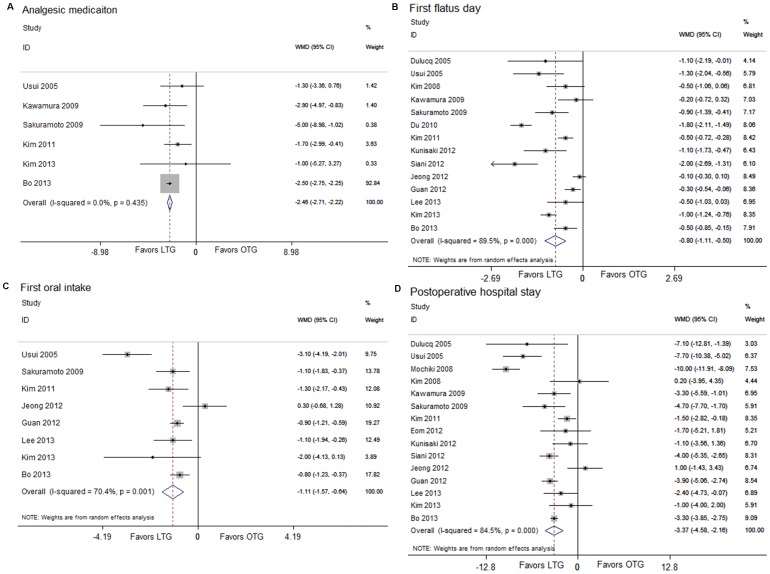
Analysis comparing (A) analgesic medication, (B) first flatus day, (C) first oral intake, and (D) postoperative hospital stay.

The postoperative pain patients suffered was evaluated by counting the times of the analgesics use. Patients receiving the laparoscopic procedure used fewer analgesics (WMD, −2.46; 95% CI, −2.71, −2.22; *P*<0.001) (Fig 3A). The outcomes also favored LTG in first flatus day (WMD, −0.80; 95% CI, −1.11, −0.50; *P*<0.001) (Fig 3B) and first oral intake (WMD, −1.11; 95% CI, −1.57, −0.64; *P*<0.001) (Fig 3C), which indicated a quicker recovery of the bowl function. Moreover, postoperative hospital day was 3.37 days shorter for LTG patients (WMD, −3.37; 95% CI, −4.58, −2.16; *P*<0.001) (Fig 3D).

### Morbidity, hospital mortality and long-term survival

**Figure 4:**
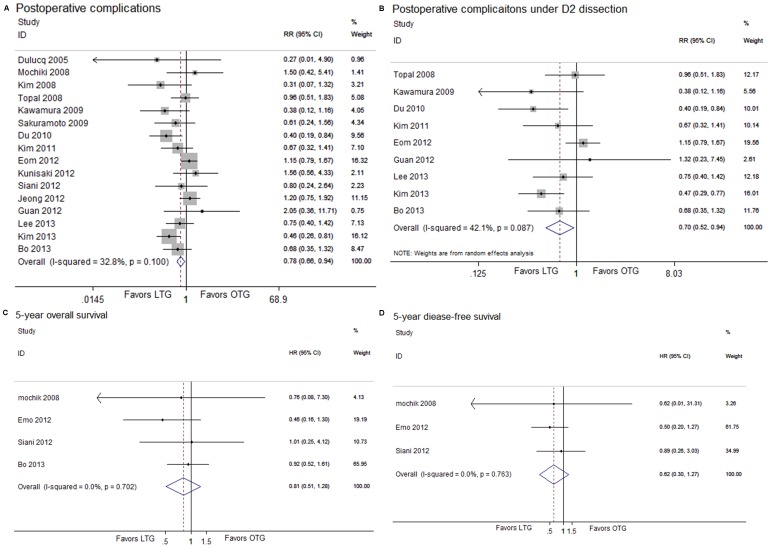
Analysis comparing (A) postoperative complications, (B) postoperative complications under D2 dissection, (C) 5-year OS and (D) 5-year DFS.

In the subcategory analysis of postoperative complications, patients in LTG group showed less wound infection (RR, 0.35; 95% CI, 0.20, 0.61; *P*<0.001). No statistical differences were found in anastomotic leakage, anastomotic stenosis, postoperative ileus, pneumonia, pancreatitis, intra-abdominal abscess and adhesive bowel obstructions between the two groups (Table 5). The overall postoperative morbidity was lower for LTG than OTG (RR, 0.78; 95% CI, 0.66, 0.94; *P* = 0.007) (Fig 4A), and the reduction of postoperative morbidity was also observed in the patients under LTG with D2 dissection when compared with patients under OTG with D2 dissection (RR, 0.70; 95% CI, 0.52, 0.94; *P* = 0.017) (Fig 4B). There were no significant differences in hospital mortality (RR, 0.94; 95% CI, 0.31, 2.82; *P* = 0.910; D2 dissection subgroup: RR, 0.57; 95% CI, 0.11, 3.09; *P* = 0.513). Six articles reported the 5-year OS of both procedures. One article directly provided the HR and the 95% CI for OS [31]. We calculated the HR and their 95% CIs from three articles [24], [34], [39] by using the published methodology. And we could not extract the HR and 95% CIs from the rest two studies due to the lack of information [33], [39]. Then, we pooled the data together, and found a favoring trend to LTG with no statistical difference (HR, 0.81; 95% CI, 0.51, 1.28; *P* = 0.360) (Fig 4C). The 5-year DFS was extracted from three studies and analyzed [24], [31], [34]. The DFS in LTG was similar with that in OTG (HR, 0.62; 95% CI, 0.30, 1.27; *P* = 0.191) (Fig 4D).

**Table 5:** Subcategory of postoperative complications comparing LTG with OTG.

		Test for Overall Effect		Test for Heterogeneity	
Items	RR 95% CI	*Z*	*P*	*I* ^2^	*P*
Anastomotic leakage	1.18 (0.61, 2.26)	0.48	0.629	<0.1%	0.656
Anastomotic stenosis	1.29 (0.72, 2.30)	0.85	0.394	<0.1%	0.839
Wound infection	0.35 (0.20, 0.61)	3.70	**<0.001**	<0.1%	0.822
Postoperative ileus	0.71 (0.28, 1.78)	0.73	0.463	<0.1%	0.949
Postoperative pneumonia	0.59 (0.29, 1.18)	1.50	0.133	<0.1%	0.955
Pancreatitis	0.56 (0.18, 1.70)	1.02	0.310	<0.1%	0.968
Intra-abdominal abscess	0.55 (0.29, 1.03)	1.87	0.062	7.4%	0.369
Adhesive bowel obstructions	0.73 (0.36, 1.48)	0.86	0.388	<0.1%	0.681

CI, confidence interval; LTG, laparoscopic total gastrectomy; OTG, open total gastrectomy; RR, relative risks; WMD, weighed mean difference; data in bold, significant *P*-value.

### Meta-regression

According to Cochrane Handbook, when a meta-analysis contains fewer than ten studies, meta-regression should generally not be considered. Therefore, we just examined the outcome variables with high heterogeneity, which included more than ten studies, in a meta-regression model. The analyses indicated that study quality, country of patients, sample size, and lymph nodes dissection were significant sources of heterogeneity (Table 6).

**Table 6:** Meta-regression analysis.

Variable	Coefficient	Standard error	*P* value	95% CI
**Operative time**				
Study quality	24.784	27.306	0.388	−36.986 to 86.553
Year of publication	−39.016	26.020	0.168	−97.877 to 19.846
Country of patients	−31.149	35.156	0.399	−110.678 to 48.379
Sample size	7.435	30.133	0.811	−60.732 to 75.601
Stage of gastric cancer	15.702	18.197	0.411	−25.463 to 56.868
Lymph node dissection	34.537	21.836	0.148	−14.861 to 83.936
**Blood loss**				
Study quality	−118.080	58.824	0.091	−262.017 to 25.858
Year of publication	−44.325	61.162	0.496	−193.983 to 105.332
Country of patients	−2.482	76.190	0.975	−188.911 to 183.947
Sample size	220.280	56.992	0.008	80.827 to 359.734
Stage of gastric cancer	77.672	43.096	0.122	−27.780 to 183.125
Lymph node dissection	−61.135	41.344	0.190	−162.300 to 40.030
**Harvested lymph nodes**				
Study quality	4.231	1.620	0.031	0.496 to 7.965
Year of publication	−0.933	1.687	0.595	−4.823 to 2.956
Country of patients	−1.116	3.720	0.772	−9.694 to 7.462
Sample size	−1.677	1.963	0.418	−6.203 to 2.850
Stage of gastric cancer	1.402	1.251	0.295	−1.482 to 4.287
Lymph node dissection	3.421	1.763	0.088	−0.643 to 7.486
**First flatus day**				
Study quality	−0.450	0.435	0.336	−1.480 to 0.579
Year of publication	0.025	0.439	0.956	−1.013 to 1.063
Country of patients	−1.169	0.621	0.102	−2.637 to 0.299
Sample size	0.109	0.437	0.810	−0.925 to 1.143
Stage of gastric cancer	0.198	0.334	0.572	−0.592 to 0.988
Lymph node dissection	−0.107	0.336	0.759	−0.901 to 0.687
**Postoperative hospital stay**				
Study quality	−2.116	0.659	0.015	−3.675 to −0.557
Year of publication	0.224	0.911	0.813	−1.931 to 2.379
Country of patients	−2.529	0.942	0.031	−4.757 to −0.301
Sample size	2.744	0.817	0.012	0.811 to 4.676
Stage of gastric cancer	0.036	0.624	0.956	−1.440 to 1.512
Lymph node dissection	2.480	0.715	0.010	0.790 to 4.170

Data in bold, significant *P*-value.

### Subgroup-analysis

As shown in Table 7, subgroup-analyses were performed by the sources of heterogeneity (study quality, country of patients and sample size), and the study characteristics we cared about (stage of gastric cancer). Because the extent of lymph node dissection is an important factor that may affect our judgment about the safety and feasibility of laparoscopic surgery, we had described the outcomes of LTG with D2 dissection in the main results above. In operative time, no decreasing trend was found in the studies with more than 50 LTG cases, and even more time was used (<50 cases: 42.97 min; ≥50 cases: 52.34 min). In blood loss, we also did not observe the statistical differences between LTG and OTG in the studies with larger sample size (≥50 cases: WMD, −82.46; 95% CI, −166.23, 1.31; *P* = 0.054). In the number of harvested lymph nodes, more lymph nodes were retrieved under OTG in the high score studies (≥8 scores: WMD, 2.56; 95% CI, 0.52, 4.59; *P* = 0.014). The fewer postoperative complications were only found in studies of AGC and articles with <8 scores (AGC: RR, 0.50; 95% CI, 0.35, 0.73; *P*<0.001; <8 scores: RR, 0.78; 95% CI, 0.61, 0.99; *P*. = 0.043). The rest outcomes remained unchanged in the subgroups.

**Table 7:** Subgroup-analyses by stage of gastric cancer, study quality, sample size and country of patients.

		Sample Size			Test for Overall Effect		Test for Heterogeneity	
Items	n^a^	LTG	OTG	RR or WMD 95% CI	*Z*	*P*	*I* ^2^	*P*
**Operative time**								
EGC	5	249	547	29.06 (4.32, 53.79)	2.30	**0.021**	94.0%	**<0.001**
AGC	3	338	418	39.93 (7.46, 72.41)	2.41	**0.016**	92.4%	**<0.001**
<8 scores	12	576	705	36.15 (19.99, 52.32)	4.38	**<0.001**	91.4%	**<0.001**
≥8 scores	5	379	653	68.38 (52.40, 84.37)	8.38	**<0.001**	70.4%	**0.009**
<50 cases	10	282	293	42.97 (21.69, 64.25)	3.96	**<0.001**	91.6%	**<0.001**
≥50 cases	7	673	1065	52.34 (29.69, 75.08)	4.51	**<0.001**	93.7%	**<0.001**
Western patients	3	71	58	28.02 (17.94, 38.09)	5.45	**<0.001**	<0.1%	0.627
**Blood loss**								
EGC	4	149	199	−242.79 (−445.19, −40.39)	2.35	**0.019**	97.8%	**<0.001**
AGC	2	199	211	−169.87 (−195.48, −144.27)	13.00	**<0.001**	<0.1%	0.417
<8 scores	10	410	465	−189.98 (−291.10, −88.86)	3.68	**<0.001**	97.0%	**<0.001**
≥8 scores	4	279	305	−152.87 (−240.92, −64.82)	3.40	**0.001**	93.0%	**<0.001**
<50 cases	9	255	260	−240.34 (−320.75, −159.93)	5.86	**<0.001**	92.7%	**<0.001**
≥50 cases	5	434	510	−82.46 (−166.23, 1.31)	1.93	0.054	94.9%	**<0.001**
Western patients	3	71	58	−150.10 (−281.84, −18.37)	2.23	**0.026**	77.4%	**0.012**
**Harvested lymph nodes**								
EGC	5	249	547	1.76 (−3.95, 7.46)	0.60	0.546	87.0%	**<0.001**
AGC	3	338	418	0.86 (−1.44, 3.16)	0.73	0.463	46.8%	0.152
<8 scores	11	538	683	1.75 (−1.72, 5.22)	0.99	0.323	79.6%	**<0.001**
≥8 scores	5	379	653	2.56 (0.52, 4.59)	2.46	**0.014**	<0.1%	0.640
<50 cases	9	244	271	3.44 (−0.38, 7.26)	1.76	0.078	71.6%	**<0.001**
≥50 cases	7	673	1065	1.15 (−1.06, 3.36)	1.02	0.308	43.9%	**0.098**
Western patients	2	33	36	0.56 (−6.16, 7.28)	0.16	0.870	42.0%	0.189
**Proximal resection margin**								
EGC	2	163	475	−0.17 (−0.35, 0.01)	1.85	0.064	<0.1%	0.793
AGC	2	256	324	−0.04 (−0.97, 0.89)	0.09	0.932	64.3%	**0.094**
<8 scores	3	243	390	−0.04 (−0.32, 0.24)	0.29	0.769	37.4%	0.203
≥8 scores	2	217	465	−0.008 (−0.69, 0.67)	0.02	0.982	80.0%	**0.025**
<50 cases	1	41	56	/	/	/	/	/
≥50 cases	4	419	799	−0.19 (−0.69, 0.32)	0.74	0.462	67.9%	**0.025**
Western patients	0	/	/	/	/	/	/	/
**Analgesic requirements**								
EGC	3	129	181	−1.87 (−2.84, −0.91)	3.80	**<0.001**	<0.1%	0.519
AGC	2	256	324	−2.50 (−2.75, −2.24)	19.26	**<0.001**	<0.1%	0.491
<8 scores	4	268	388	−1.83 (−2.77, −0.89)	3.81	**<0.001**	<0.1%	0.691
≥8 scores	2	147	161	−2.51 (−2.76, −2.26)	19.38	**<0.001**	<0.1%	0.219
<50 cases	3	98	96	−2.44 (−3.81, −1.07)	3.49	**<0.001**	32.4%	0.228
≥50 cases	3	419	451	−2.47 (−2.71, −2.22)	19.40	**<0.001**	<0.1%	0.390
Western patients	0	/	/	/	/	/	/	/
**First flatus day**								
EGC	3	129	181	−0.58 (−1.04, −0.12)	2.49	**0.013**	65.1%	**0.057**
AGC	3	338	418	−1.10 (−1.78, −0.42)	3.17	**0.002**	93.6%	**<0.001**
<8 scores	10	518	665	−0.72 (−1.04, −0.41)	4.56	**<0.001**	85.7%	**<0.001**
≥8 scores	4	279	305	−0.94 (−1.64, −0.23)	2.61	**0.009**	91.6%	**<0.001**
<50 cases	8	224	253	−0.87 (−1.28, −0.45)	4.11	**<0.001**	78.5%	**<0.001**
≥50 cases	6	513	717	−0.73 (−1.22, −0.25)	2.97	**0.003**	94.5%	**<0.001**
Western patients	2	33	36	−1.75 (−2.33, −1.17)	5.89	**<0.001**	46.5%	0.172
**First oral intake**								
EGC	2	83	146	−2.17 (−3.93, −0.41)	2.41	**0.016**	84.3%	**0.012**
AGC	2	256	324	−0.85 (−1.27, −0.43)	3.94	**<0.001**	14.7%	0.279
<8 scores	5	385	531	−1.30 (−2.23, −0.36)	2.71	**0.007**	82.4%	**<0.001**
≥8 scores	3	197	211	−0.91 (−1.25, −0.57)	5.28	**<0.001**	<0.1%	0.703
<50 cases	3	91	119	−1.58 (−2.63, −0.53)	2.95	**0.003**	86.2%	**0.001**
≥50 cases	5	491	623	−0.84 (−1.38, −0.30)	3.03	**0.002**	49.0%	**0.098**
Western patients	0	/	/	/	/	/	/	/
**Postoperative hospital stay**								
EGC	5	249	547	−4.87 (−8.60, −1.14)	2.56	**0.010**	93.4%	**<0.001**
AGC	2	256	324	−3.23 (−3.77, −2.68)	11.64	**<0.001**	54.3%	0.139
<8 scores	11	538	683	−3.46 (−5.31, −1.62)	3.68	**<0.001**	88.6%	**<0.001**
≥8 scores	4	297	559	−3.26 (−3.79, −2.74)	12.23	**<0.001**	<0.1%	0.534
<50 cases	9	244	271	−4.65 (−6.52, −2.75)	4.80	**<0.001**	84.9%	**<0.001**
≥50 cases	6	591	971	−1.70 (−3.08, −0.31)	2.40	**0.016**	72.4%	**0.003**
Western patients	2	33	36	−4.16 (−5.48, −2.85)	6.21	**<0.001**	6.7%	0.300
**Postoperative complications**								
EGC	4	229	538	0.94 (0.69, 1.28)	0.40	0.689	38.8%	0.179
AGC	3	338	418	0.50 (0.35, 0.73)	3.68	**<0.001**	<0.1%	0.523
<8 scores	11	556	686	0.78 (0.61, 0.99)	2.03	**0.043**	32.5%	0.139
≥8 scores	5	379	653	0.79 (0.61, 1.03)	1.78	0.076	46.6%	0.112
<50 cases	9	262	274	0.79 (0.55, 1.13)	1.30	0.192	3.2%	0.408
≥50 cases	7	673	1065	0.75 (0.54, 1.04)	1.72	0.085	57.3%	**0.029**
Western patients	3	71	58	0.84 (0.48, 1.47)	0.61	0.540	<0.1%	0.675
**Hospital mortality**								
EGC	3	166	401	1.60 (0.27, 9.64)	0.51	0.609	<0.1%	0.665
AGC	2	221	301	0.23 (0.01, 4.70)	0.96	0.339	/	/
<8 scores	6	360	418	1.42 (0.31, 6.46)	0.46	0.649	<0.1%	0.674
≥8 scores	4	262	536	0.57 (0.11, 3.09)	0.65	0.513	<0.1%	0.392
<50 cases	5	129	133	1.10 (0.15, 8.09)	0.10	0.923	<0.1%	0.420
≥50 cases	5	493	821	0.88 (0.23, 3.28)	0.20	0.843	<0.1%	0.528
Western patients	3	71	58	0.44 (0.02, 9.69)	0.52	0.606	/	/

CI, confidence interval; LTG, laparoscopic total gastrectomy; OTG, open total gastrectomy; RR, relative risks; WMD, weighed mean difference; EGC, early gastric cancer; AGC; advanced gastric cancer; data in bold, significant *P*-value.

a Number of comparisons.

### Sensitivity and Publication Bias

**Figure 5:**
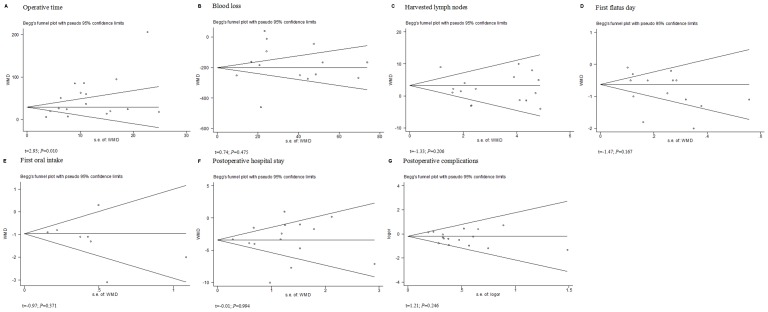
Funnel plots of each outcome. A, operative time; B, blood loss; C, harvested lymph nodes; D, first flatus day; E, first oral intake; F, hospital stay; G, postoperative complications.

We used the funnel plots and Egger's linear regression test to detect publication bias for each result. When the number of studies was small, there was a limitation in this test. So the funnel plots of proximal resection margin, analgesic medication, hospital mortality, 5-year OS and DFS, were not showed. Eventually, seven funnel plots were constructed for the outcomes we most cared about. The symmetry of most outcomes on the whole was observed. All the outcomes showed no significant publication bias (P>0.05) except operative time (t = 2.93; *P* = 0.010) (Fig 5). Galbraith plot was used to find which articles were the contributors to heterogeneity. Then we excluded these articles and analyzed the pooling data of the rest. The same conclusions were found.

## Discussion

According to “Gastric Cancer Treatment Guidelines in Japan, 2010”, total gastrectomy is used in radical resection of proximal and middle third gastric cancer. Laparoscopic surgery is recommended as a treatment for early gastric cancer and clinical research. Patient's preference and surgeon's suggestion may affect the choice of operation type. And cosmetic result, cost, recovery and pain are the major factors the patients care about [5], [40]–[43]. Recently, a meta-analysis has shown the superiority of LTG to OTG [13], however, the oncological outcome and long-term outcomes are still needed to evaluate. With the development of laparoscopic technique, the number of LTG use is increasing, and seven additional articles that compared the LTG with OTG have been published. Therefore, we performed this updated meta-analysis to estimate the value of LTG.

The randomized controlled trials (RCTs) are our first choice for the high quality of the outcomes. But no RCTs focusing on this subject were found. Eighteen non-randomized comparative cohort studies were selected. In order to get convincible results from articles, NOS was used to assess the quality of the studies and one low-quality study was excluded. Then, we compared the clinical characteristics between the two groups and no statistical differences were found in age, sex, ASA and pTNM stages except BMI which was lower in LTG groups. This finding indicated that selection bias might exist among the studies. Surgeons might prefer to perform LTG on thinner people and our conclusion might bias to laparoscopic procedures. Considering that more than half of the articles reported the gastrectomy with D2 dissection, which was preferred by Asian surgeons, the fact that most studies we collected were from eastern countries was reasonable. Compared with patients in western countries, Asian patients are younger, slimmer and healthier [44]. These factors were associated with better postoperative outcomes after open gastrectomy [45]–[47]. So there might be a bias to Asian people in our analysis. Thus, we assessed the outcomes of the western patients in the subgroup, and the similar results were found expect the lower postoperative morbidity. Because only three studies were involved, more studies were needed. Publication bias for each variable was detected by using funnel plots and Egger's linear regression test, and no significant publication bias of each outcome was found except operative time. In general, because the quality of all the studies was assured by NOS and most of the clinical characteristics were matched, the two groups were comparable.

Due to the lack of tactile sensation, narrow operating field, complicated vascular structure in the splenic hilum, and the advanced techniques for systemic lymph node dissection, LTG was regarded as a time-consuming procedure. Haverkamp *et al.* also reported a longer duration of operative time in LTG groups [13]. Early learning curve, the familiarity with the laparoscopic system and the cooperation of the whole therapeutic team were thought as the factors that influence the operative time [48]. According to the studies of learning curve in LDG, 40–60 cases are needed [48]–[50]. Thus, we used 50 LTG cases as a cut point and performed the subgroup analysis. However, no reduction in LTG operative time was observed and even more time was used. Our result suggested that learning curve was not the main cause of the longer time, which was consistent with the conclusion that even with experienced gastrointestinal and laparoscopic surgeons, laparoscopic gastrectomy is still a time-consuming procedure [51]. Thus, the complexity of LTG and the shortage of the laparoscopic equipments might be the main reason. With the technological improvement and the development of the instruments, the reduction of the operating time has been observed in LDG [51], [52]. It is believed that the time for LTG will decrease in the future.

In spite of the longer operative time, a significant decrease of blood loss for laparoscopic approach was found compared with open procedure, which indicated fewer transfusions during the operation. The enlarged laparoscopic surgical field with the advantage of better vessel exposing and identifying contributed to this outcome, which is also attributed to the use of special instruments, such as the ultrasonic scalpel and ligatures [53]. The recovery of the patients was affected by both the amount of blood loss and the requirement of transfusion [54], [55]. The less blood loss can reduce the risk of acute or late adverse effects such as acute lung injury, volume overload, hypothermia, etc. However, in the subgroup analysis, this benefit was not observed in the studies with higher scores, although there was a tendency favoring LTG. Further validations are still required.

The length of resection margin can influence the rate of tumor-free margins. Thus, whether LTG can resect the similar length as OTG is very important for the oncological safety. Because the resection of proximal stomach is more difficult than the duodenal resection, most of the involved articles just provided the length of proximal resection margin. No statistical difference of this subject was found, which indicated the similar ability of proximal resection between LTG and OTG. This finding can also explain the similar positive rate of resection margin reported by three studies [22], [26], [29].

The results were significantly favoring for LTG in the use of analgesics, first flatus day, first oral intake and the length of hospital stay. In this analysis, the times of the analgesics use were extracted to evaluate the postoperative pain, because pain-feeling is hard to measure for its subjectivity. Less pain during recovery is most likely caused by the minimal invasiveness of LTG and it suggests earlier recovery and better quality of life. Earlier passage of flatus represents a quicker recovery of bowel function, which has a direct impact on earlier resumption of oral intake and earlier discharge from hospital. Minimal gastrointestinal interference and the use of small incision can explain all the advantages above, and can also decrease surgical stress, therefore reduce the generalized inflammatory reaction, leading to a reduction of postoperative morbidity.

The extent of lymph node dissection is a critical factor for oncological adequacy. D1 dissection is now accepted as a standard treatment for selected patients with early gastric cancer. Debate about whether D2 dissection for curable advanced gastric cancer is superior to D1 dissection still exists. The more complexity and invasiveness of D2 dissection are thought to increase the postoperative complications and mortality. Whereas, D2 dissection is possible to remove more positive nodes than D1 dissection, which is necessary to minimize stage migration[56]. The western scholars preferred the D1 dissection because some European reports have shown that D2 dissection had no survival advantages but with a higher operative morbidity and mortality rates [57]–[59]. On the other hand, the high incidence of gastric caner makes Asian surgeons more familiar with gastrectomy, which results in better understanding of the indications of D2 dissection and surgical technique. Therefore, D2 dissection is recommended by many eastern surgeons for the improved long-term survival [60]–[62]. According to “Gastric Cancer Treatment Guidelines in Japan, 2010”, standard radical gastrectomy was even defined as: more than two-thirds of proximal, distal, or total gastrectomy associated with D2 dissection. And more recent reports have demonstrated that European surgeons can be trained to perform D2 dissection for selected western patients with better postoperative outcomes and a survival benefit [63]–[66]. Thus, D2 dissection is felt to be a more appropriate treatment for patients with advanced disease at present. Recently, LTG with D2 dissection (LTGD2) has been reported to be feasible in the hands of experienced surgeons [67]. Nevertheless, the difficulty of performing LTGD2 may affect the benefits of laparoscopic surgery. Considering our analysis including nine studies of D2 dissection, it is necessary to analyze the number of harvested lymph nodes, postoperative morbidity and hospital mortality after the gastrectomy with D2 dissection in the subgroup.

The number of harvested lymph nodes is used to evaluate the oncological adequacy. According to UICC (Union for International Cancer Control), the removal of at least fifteen lymph nodes is beneficial for pathological examination. The mean number of lymph nodes retrieved by LTG was adequate in all studies. In Haverkamp *et al.*'s analysis, more lymph nodes were harvested by OTG than by LTG, though no significant difference was found. In our analysis, we discovered the same results, which indicated the similar ability of lymph nodes clearance between the two procedures. Considering the difficulty of clearing the lymph nodes around the splenic artery and hilus of the spleen under LTGD2, the measurement of adequate lymphadenectomy by LTGD2 is necessary. The similar clearance of lymph nodes was also observed. In D2 dissection of total gastrectomy, splenectomy is performed to resect lymph nodes around splenic artery (No. 11) and hlium (No. 10). But splenectomy was reported associated with higher postoperative morbidity and mortality without survival benefits [68]–[70]. According to the recent Guidelines from the National Comprehensive Cancer Network (NCCN), splenectomy is recommended only when spleen or hilum is involved, and modified D2 dissection (without pancreatectomy and splenectomy) is suggested performed by experienced surgeons in high-volume centers for patients with localized resectable cancer. Nine studies in our meta-analysis reported the performance of LTG with modified D2 dissection, and eight of them showed the number of harvested lymph nodes. All the studies pronounced the dissection of No. 11 lymph nodes and four studies did not mention the retrieval of No. 10 lymph nodes. Two studies can be explained by the low incidence of hilar lymph nodes metastasis in early gastric cancer [27], [31]. The other two did not show us the reason why they performed the LTGD2 for advanced gastric cancer without the clearance of No. 10 lymph nodes [29], [39]. The difficulty of splenic hilar lymphadenectomy by using spleen-preserving LTG due to the complicated anatomic relationship between lymph nodes and vessels around splenic hilum may explain it. Therefore, we also assessed the effect of No. 10 lymph nodes dissection on the number of harvested lymph nodes. And no statistical difference was found regardless of the removal of No. 10 lymph nodes dissection between LTG and OTG. All above demonstrated that LTG had a similar ability of lymph nodes clearance compared with OTG, but there is one important thing we should notice. In the studies with higher scores, OTG was believed to retrieve more lymph nodes. Combing with the result that a favoring trend towards OTG was found in the other subgroups, further validation of this subject are still needed.

The postoperative morbidity is an important outcome to assess the safety of the operation type. In the subcategory analysis, reduced wound infection in LTG group was found due to the scattered trocar incisions and contractible sample-extraction incision. The same technique of digestive reconstruction in both procedures could explain the similar incidence of anastomotic leakage and stenosis. The minimal invasiveness of laparoscopic surgery could reduce the intervention to microenvironment of abdominal cavity and injury of intestinal serous membrane, which was thought being able to decrease the occurrence of postoperative ileus, pneumonia, pancreatitis, intra-abdominal abscess and adhesive bowel obstructions. In our analysis, we did not observe any statistical difference in these aspects, but a favorable trend in LTG was found. The relative small sample size in the subcategory might be the reason. When we pooled the data together, the patients undergoing LTG were associated with a significant reduction of total postoperative complications. And the same result was also found in the subgroup of D2 dissection. But in the other subgroups, only the studies of AGC or articles with <8 scores showed the fewer postoperative complications in LTG groups. Considering that low heterogeneity was found in the overall result and the rest subgroups showed a tendency towards LTG, this result can be explained by a relative small sample size in subgroups, which did not have enough volume to show the statistical difference. Therefore, the lower postoperative morbidity in LTG group should be reliable.

Our analysis revealed that there was no significant difference in hospital mortality between the two groups. And in the subgroup of D2 dissection, the same conclusion was found. These suggested the equivalent short-term prognosis between LTG and OTG. Long-term outcome is the most important factor used for evaluating the oncological safety of one surgery. The majority of recurrences occurs during the first two years after surgery [71], so we used two years as the qualification for NOS to assess the adequate follow-up period of each study. We extracted the 5-year OS and DFS from all available articles. Although the results favored LTG, there were no statistical differences indeed. Combining with the similar lymph nodes clearance and proximal resection, we could say LTG has the similar oncological safety and adequacy with OTG in some extent. However, the relative small sample size in long-term outcomes made our conclusion not convincible enough. More studies focusing on this subject are still needed.

In conclusion, with the less blood loss, quicker postoperative recovery, reduced postoperative morbidity, and similar oncological safety, LTG is a feasible and safe surgery for gastric cancer. LTG can be performed as an alternative to OTG for selected patients by experienced surgeons in high-volume centers. However, well-designed RCTs in multicenter and the comparative studies of long-term outcomes are still needed for further validation.

## Supporting Information

Checklist S1 PRISMA Checklist.
(DOC)
